# Guiding the design of SARS-CoV-2 genomic surveillance by estimating the resolution of outbreak detection

**DOI:** 10.3389/fpubh.2022.1004201

**Published:** 2022-10-05

**Authors:** Carl J. E. Suster, Alicia Arnott, Grace Blackwell, Mailie Gall, Jenny Draper, Elena Martinez, Alexander P. Drew, Rebecca J. Rockett, Sharon C.-A. Chen, Jen Kok, Dominic E. Dwyer, Vitali Sintchenko

**Affiliations:** ^1^Centre for Infectious Diseases and Microbiology Public Health, Westmead Hospital, Westmead, NSW, Australia; ^2^Sydney Institute for Infectious Diseases, The University of Sydney, Westmead, NSW, Australia; ^3^Centre for Infectious Diseases and Microbiology Laboratory Services, Institute of Clinical Pathology and Medical Research, NSW Health Pathology, Westmead, NSW, Australia

**Keywords:** genomic surveillance, public health, pathogen genomics, SARS-CoV-2, outbreak detection

## Abstract

Genomic surveillance of SARS-CoV-2 has been essential to inform public health response to outbreaks. The high incidence of infection has resulted in a smaller proportion of cases undergoing whole genome sequencing due to finite resources. We present a framework for estimating the impact of reduced depths of genomic surveillance on the resolution of outbreaks, based on a clustering approach using pairwise genetic and temporal distances. We apply the framework to simulated outbreak data to show that outbreaks are detected less frequently when fewer cases are subjected to whole genome sequencing. The impact of sequencing fewer cases depends on the size of the outbreaks, and on the genetic and temporal similarity of the index cases of the outbreaks. We also apply the framework to an outbreak of the SARS-CoV-2 Delta variant in New South Wales, Australia. We find that the detection of clusters in the outbreak would have been delayed if fewer cases had been sequenced. Existing recommendations for genomic surveillance estimate the minimum number of cases to sequence in order to detect and monitor new virus variants, assuming representative sampling of cases. Our method instead measures the resolution of clustering, which is important for genomic epidemiology, and accommodates sampling biases.

## 1. Introduction

Whole genome sequencing (WGS) has become an integral component of the public health response to communicable disease outbreaks, particularly during the severe acute respiratory syndrome coronavirus 2 (SARS-CoV-2) pandemic (1–3). In the context of the pandemic, a number of institutions have published guidelines for genomic surveillance (4–6). These recognize that for maximum utility, population level genomic surveillance should consist of both representative sampling of cases in a community to monitor the emergence and proportions of circulating SARS-CoV-2 variants, together with targeted sampling to characterize particular variants or genomes from cohorts of public health interest. In addition, to assist with outbreak investigation, WGS has been used to resolve contentious links between cases and to identify links in the absence of epidemiological evidence, thereby improving the accuracy of case clustering (7). Accurate clustering enables understanding of the transmission characteristics of the virus including the geospatial dynamics and the dominant settings of transmission both in the community and in healthcare and other facilities (8). This in turn enables appropriate public health measures to be implemented.

In order for a genome sequence to be generated from an infection with the virus, a suitable specimen must first be referred to a sequencing laboratory, and that laboratory must allocate resources to sequence and analyze the sample. The degree to which genomic surveillance is representative is therefore sensitive to case identification (9–11), deployment of diagnostic testing (12), timeliness of referral to sequencing laboratories, viral load of specimens, and sequencing throughput. Bias can be introduced unintentionally at each point in this process by characteristics of a new virus variant, inequities in access to diagnostic testing, and logistical impediments. When community incidence is high, targeted sequencing induces intentional biases by typically referring or sequencing only specimens associated with cases from specific settings or cohorts (13, 14). Targeted sequencing may result in the loss of crucial information or context provided by representative sequencing.

In Australia, and indeed around the world, high incidence of infection driven by the Delta and Omicron SARS-CoV-2 variants has made ambitions to subject all cases to WGS untenable, including in regions which had previously sequenced the majority of cases. Public health units and laboratories have been forced to adopt targeted sampling strategies that mitigate selection bias and latency within their logistical and financial constraints. Furthermore, laboratory-based diagnostic tests have been restricted to higher-risk settings with a greater reliance on self-administered rapid antigen tests for case detection, meaning that fewer samples are available for sequencing (15). This has resulted in a growing interest amongst governments and public health agencies in determining the minimum depth of genomic surveillance necessary to achieve their desired public health outcomes. The European Centre for Disease Prevention and Control (ECDC) published guidelines based on statistical considerations for the minimum number of sequences necessary to detect new variants or changes in the proportion of variants assuming representative sampling (5). Recent work has sought to improve the practical utility of these guidelines by simulating or modeling the steps in between infection with SARS-CoV-2 and genomic characterization (16, 17). These approaches are important because they explicitly account for the effect of some sources of selection bias.

Methods that could inform the rational design of genomic surveillance in the context of variable disease incidence and needs of public health response remain underdeveloped. In particular, existing frameworks focus on detecting and monitoring variants, but do not provide a means to quantify the effect of reduced depth of genomic surveillance on the resolution of case clustering or phylogenetic analysis. The sensitivity of phylogenetic analyzes to sampling biases is recognized (18, 19), and has previously been addressed by downsampling genomic data to remove over-represented categories of sequences based on available epidemiological data (20) or by explicitly incorporating biases into models (21, 22). Such models have had important roles in distinguishing multiple importations from cryptic transmission, but can be expensive to operate both computationally and in terms of the necessary expertise. A framework that models the effect of potentially biased selection strategies on genomic epidemiology in terms of cluster identification, to enable development of effective genomic surveillance systems, would be of great public health utility.

We present here a method for estimating the probability of recognizing representative clusters from outbreaks of different sizes and at different stages in their progression, given assumptions about the outbreak parameters. We demonstrate the approach using synthetic data for which the true lineage of cases is known, as well as genomic surveillance data from outbreaks of SARS-CoV-2 in New South Wales (NSW), Australia. The model described herein could be applied to any pathogen of public health importance.

## 2. Methods

### 2.1. Surveillance data and outbreak definition

Our method tests how the clustering in outbreak cases varies with the proportion of cases observed. The approach is general and can be adapted to different types of continuous data. For the purposes of this study, we assume that for each case the genome sequence of the pathogen (i.e., SARS-CoV-2 in this study) and date of specimen collection are known. For each data type (i.e., temporal distances between cases and evolutionary distance between associated pathogen genomes), we compute a pairwise distance matrix: the Hamming distance in the case of the WGS data and the usual Euclidean distance for the date of collection.

Each case must be assigned to an outbreak. In simulated data, the outbreaks correspond to separate introductions of the disease into a community, while in empirical data the outbreaks are based on clusters of cases informed by both phylogeny and contact tracing. Sample datasets of different sizes are then drawn at random from the full set to simulate lower levels of surveillance. For each of these datasets, cases are clustered using two distance-based algorithms. The generated clusters are then assessed using the known outbreak membership to determine whether each outbreak is likely to have been detected.

### 2.2. Clustering using distance thresholds

The first clustering method uses distance thresholds to associate cases with others that are plausibly connected by transmission. For each data type, a threshold distance value is selected. In sparsely sampled outbreaks, the average distances between observed cases will be larger than the actual distances between cases and their source of infection due to the unobserved missing links; the thresholds can therefore be increased to account for the estimated case reporting rate (23).

We infer an undirected graph from the data where each vertex represents a case, and an edge connects any two vertices where the pairwise distance between the two corresponding cases is not more that the respective threshold for every data type. The connected components of the graph then correspond to the clusters. This design is intended to capture the fact that if two cases are related by transmission (possibly with unobserved intermediate cases), they are likely to be similar to each other in every data type.

### 2.3. Clustering using nearest neighbors

**Figure 1:**
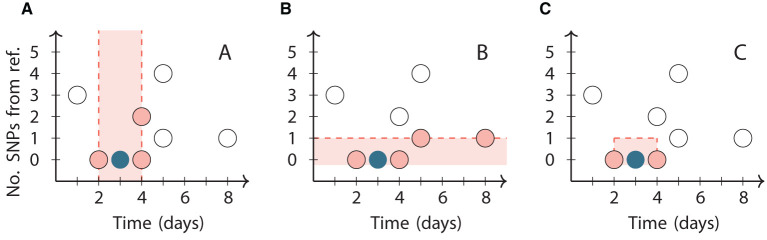
Illustration of the neighborhoods in the kNN clustering algorithm for *k* = 3 applied to a reference point (dark circle) chosen from a synthetic dataset (all circles). The vertical axis represents the number of SNPs relative to the reference point. **(A)** The third closest point in time to the reference point is 1 day from it, so its temporal neighborhood includes all three points within 1 day of it (pink circles). **(B)** The third closest genome is 1 SNP from the reference point, so its genetic neighborhood includes all four points within 1 SNP of it (pink circles). **(C)** The two points that are in both the temporal and genetic neighborhoods (pink circles) are the final neighbors of the reference point. The *k* = 4 neighborhood includes the same two points and additionally the point at day 5 with 1 SNP.

We use a second clustering method that does not depend on subjective thresholds, based on Jacques' popular *k* nearest neighbors (*k*NN) test (24). The *k*NN test works by computing the *k*-neighborhood of a case: the intersection of the set of its *k* nearest neighbors (including ties) in each data type (Figure 1). If there is an interaction between the data types as would be expected in the scenario of transmission of an infectious disease, then these *k*-neighborhoods are expected to be large.

We fix a choice of *k* and construct an undirected graph with one node for each case, and edges connecting each case to the cases in its *k*-neighborhood. The connected components of the resulting graph form the clusters. The clusters created by different choices of *k* are not independent, since each (*k*+1)-neighborhood of a case is a superset of its *k*-neighborhood. The technique can therefore generate a hierarchical clustering of the cases by varying *k*.

### 2.4. Assessing clustering

Conventional clustering analyses are typically based on pairwise genetic distances or partitions of phylogenetic trees (25, 26) with some degree of manual curation to ensure cluster plausibility and stability, and incorporation of epidemiological data (27). Our clustering approaches are intended to identify situations where a more thorough investigation would likely discover the outbreaks, while remaining completely automated and restricted to pairwise distance data. These restrictions are necessary to enable the efficient evaluation of many scenarios and testing strategies.

We compare the composition of clusters generated by the two algorithms with the known outbreak membership, and classify each cluster as either “informative” or “uninformative.” A cluster is informative when at least 80%—and no fewer than three—of its cases belong to a single outbreak. Clusters in which 20% or more of cases belong to other outbreaks are uninformative as they suggest a situation where distinct outbreaks would be challenging to distinguish in practice. Very small mixed clusters are likely to arise from coincidental proximity of cases.

**Figure 2:**
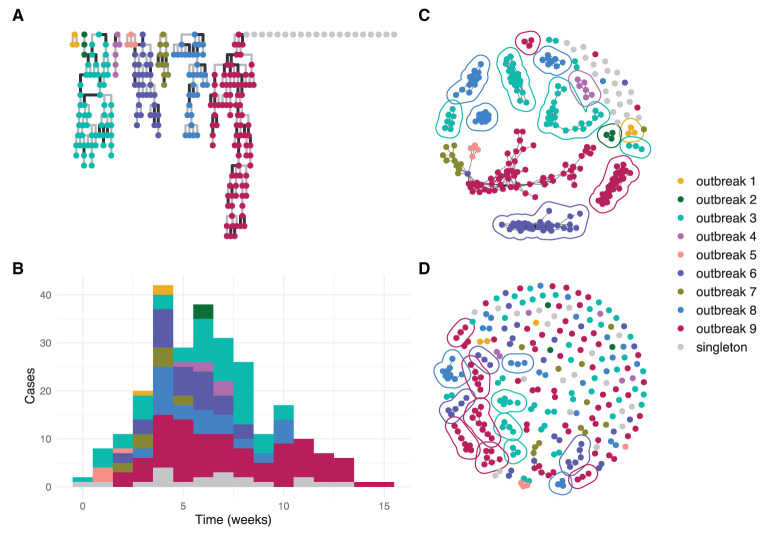
An example simulated dataset modeling several community outbreaks of SARS-CoV-2 of different sizes. Each circle represents an individual genome. **(A)** transmission trees from the branching process for each outbreak with darker lines representing transmissions introducing at least one SNP; **(B)** epidemiological curve showing cases per week by outbreak; **(C)** reconstructed clusters using the threshold method with thresholds 1 SNP and 7 days; **(D)** reconstructed clusters using the nearest neighbor method with *k* = 4. In **(C,D)**, a line encircles each informative cluster.

Figure 2 shows an example simulation and the output of the clustering algorithms. In this simulation, the threshold method (Figure 2C) generated informative clusters for most outbreaks, but the largest cluster is uninformative as in contains cases from four distinct outbreaks. The nearest neighbor method with *k* = 4 (Figure 2D) generated smaller clusters, with many of the outbreaks split across multiple informative clusters. Outbreaks 5 and 7 are the only outbreaks in the example that are not resolved by either method because they do not have at least one informative cluster.

Public health surveillance programs are of most value when new introductions are detected in real time to allow for effective interventions; discovering outbreaks retrospectively is typically of less impact. We therefore assess whether the outbreaks are resolved at three times: *t*_25%_, the day when one quarter of the outbreak's cases have been reported; *t*_50%_, the day when one half of the cases have been reported; and *t*_100%_, the final day of the cluster. For each assessment, all cases in the simulation that were reported by the specified time are provided to the clustering algorithm.

### 2.5. Simulated SARS-CoV-2 outbreaks

To simulate outbreaks, we use a simple branching process model coupled with a simple model of the pathogen genome as a list of positions with two states. Details of the simulation and its parameters are described in the Supplementary material. Simulated outbreaks are approximately consistent with reported SARS-CoV-2 parameters including its serial interval distribution, infectiousness, and mutation rate (28–30).

**Figure 3:**
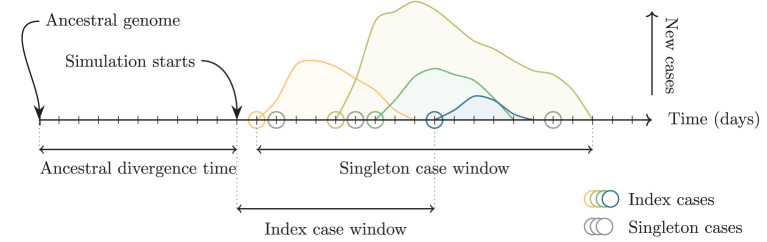
Schematic of relevant time intervals in the simulation. The ancestral divergence time is the interval between the ancestral genome and the start of the simulation. Increasing this interval increases the average genetic distance between the index cases of the outbreaks. Index cases (colored circles) are assigned dates uniformly at random on the interval defined by the index case window. Increasing this interval increases the average temporal distance and consequently also the average genetic distance. Singleton cases (gray circles) are assigned dates uniformly at random on the interval between the first and final simulated cases from any outbreak.

To simulate multiple independent importations, multiple outbreaks are created using the above procedure. The times of each outbreak are shifted so that the infection times of the index cases fall uniformly at random on a fixed time interval, the *index case window*. The genomes of each index case are taken to have descended from a common ancestor infected before the start of the simulation (the *ancestral divergence time*) and randomly acquire mutations according to the mutation rate and the time elapsed (Figure 3).

The simulation can generate outbreaks of very different sizes for the same parameters. We define three size bins with fixed capacity: four small outbreaks with 2 < *N* ≤ 10 cases, three medium outbreaks with 10 < *N* ≤ 40 cases, and two large outbreaks with 40 < *N* ≤ 180 cases. Outbreaks are simulated and discarded unless they fit into a size bin that is not already full, until all bins are filled. We then insert 20 singleton cases to simulate background cases that are not associated with any cluster. Simulations for several alternative scenarios are presented in the Supplementary material including where isolated outbreaks occur against a much larger background of unclustered cases.

### 2.6. Application to a SARS-CoV-2 Delta variant outbreak

In the state of NSW, Australia, a wave of SARS-CoV-2 infections associated with the Delta variant began in mid 2021, quickly outpacing the capacity for both contact tracing and WGS. We applied our method to data from the first 3 months of this wave to assess delays in identifying clusters in scenarios where the depth of genomic surveillance is reduced compared to what was actually undertaken.

Samples included in this study were all clinical respiratory samples that tested positive using SARS-CoV-2 reverse transcription polymerase chain reaction (RT-PCR), collected between 15 June and 19 September 2021 and referred to the Institute for Clinical Pathology and Medical Research (ICPMR) for WGS. All included samples were subsequently sequenced and assigned to the Delta lineage using the Pangolin classification system (31). Illumina WGS and bioinformatic analysis were performed according to previously published methods (7).

Single nucleotide polymorphism (SNP) distances were determined from the consensus sequences using snp-dists (https://github.com/tseemann/snp-dists version 0.7.0) after aligning to the reference sequence (RefSeq accession NC_045512.2) with nextalign [https://github.com/nextstrain/nextclade version 2.2.0 (32)] and masking the first 100 and final 200 positions. Cases were assigned genomic clusters as part of routine genomic surveillance with new cases added to an existing cluster if they differed from its index case by fewer than three SNPs.

## 3. Results

### 3.1. Simulated outbreaks

**Figure 4:**
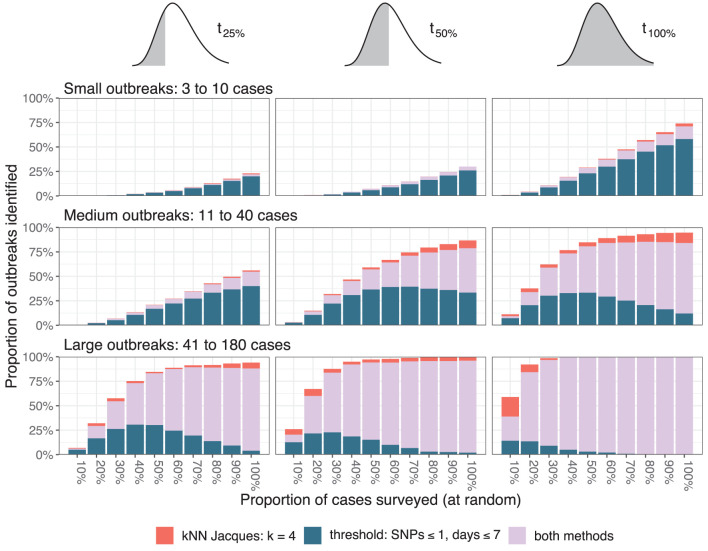
Resolution of outbreaks. The three columns correspond to the time points when the assessment occurred (after one quarter, one half, or all the cases in a cluster are reported), and the three rows correspond to the size of the outbreaks measured by the total number of simulated cases. The color of the bars indicates whether one or both clustering methods resolved the outbreaks.

The resolution of outbreak detection achieved by different depths of genomic surveillance for the baseline scenario is summarized in Figure 4. The proportion of outbreaks identified was determined by counting outbreaks with at least one informative cluster generated by either clustering method. To reduce random effects due to a particular run of the simulation, values shown were averaged over 25 independent simulations. To reduce random effects due to the choice of sample, values for every simulation were averaged over 50 independent samples at each depth of surveillance.

Twenty-five percent of small outbreaks were not identified on the day of their final case even when all cases were sequenced, and more than 75% were not identified at *t*_25%_. The proportion identified decreased linearly as the proportion sequenced was decreased. At *t*_50%_ and *t*_100%_ the proportion of medium outbreaks identified varied non-linearly with the proportion sequenced. At least 75% of large outbreaks were identified by *t*_25%_ when at least 40% of cases were sequenced. The relative importance of the two algorithms varied with the outbreak size and surveillance depth, with the nearest neighbor method failing to identify small outbreaks but successfully resolving larger outbreaks missed by the threshold method when the proportion sequenced was low.

**Figure 5:**
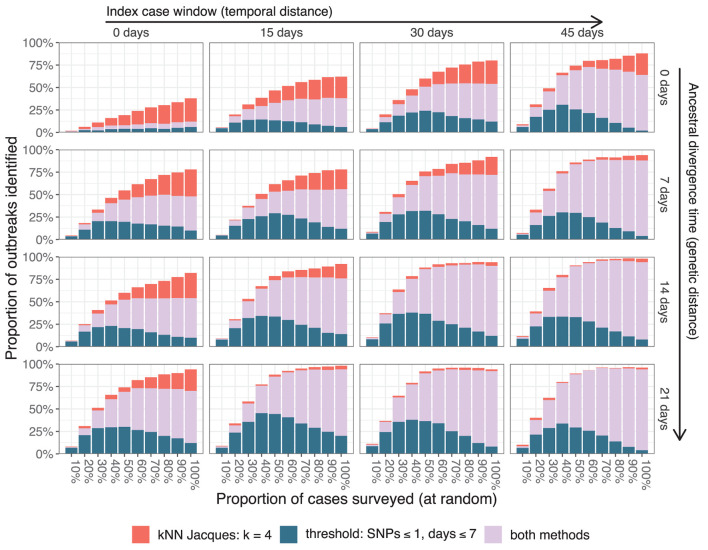
Resolution of large outbreaks at *t*_25%_ as the index case window and the ancestral divergence time are varied. The color of the bars indicates whether one or both clustering methods resolved the outbreaks.

The impact of the two major simulation parameters affecting the average distance between cases—the index case window and the ancestral divergence time—is summarized in Figure 5 for large outbreaks. The results were averaged over 25 independent simulations for every combination of parameter values and 20 independent samples from each simulation at each depth of surveillance. Simulations toward the bottom and right of Figure 5 (index case window 30 or 45 days and ancestral divergence time 14 or 21 days) had outbreaks with index cases that were typically weeks and several SNPs apart, consistent with independent importations of distinct lineages. In these simulations, almost all outbreaks were identifiable by *t*_25%_ using the threshold clustering method. Simulations toward the top and left of the figure (index case window 0 or 15 days and ancestral divergence time 0 or 7 days) had index cases occurring within a few days and where the associated pathogens often had the same sequence. These were less frequently identifiable by the threshold method.

### 3.2. Delta variant outbreak in NSW

**Figure 6:**
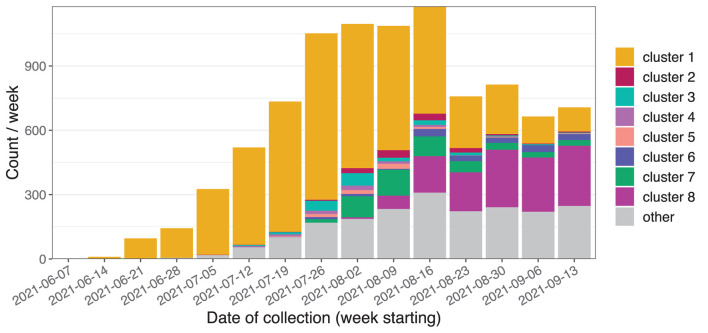
Initial weeks of the Delta variant outbreak in NSW showing sequences belonging to the Delta variant where the sample was collected between 15 June and 19 September 2021.

During the study period, 9,184 Delta variant sequences were generated (Figure 6). The initial cases were part of genomic cluster 1, and subsequently genomic clusters 2 to 8 were identified as distinct sub-clusters of cluster 1. A number of smaller sub-clusters, additional clusters, and unclustered cases were grouped into the “other” category. The median distance between cases was 5 SNPs (inter-quartile range 3–7 SNPs) and 20 days (IQR 10–34 days). Within clusters the median distances between cases ranged from one to three SNPs and from 10 to 18 days. The three largest clusters contained 4,850, 1,223, and 462 cases, respectively.

**Figure 7:**
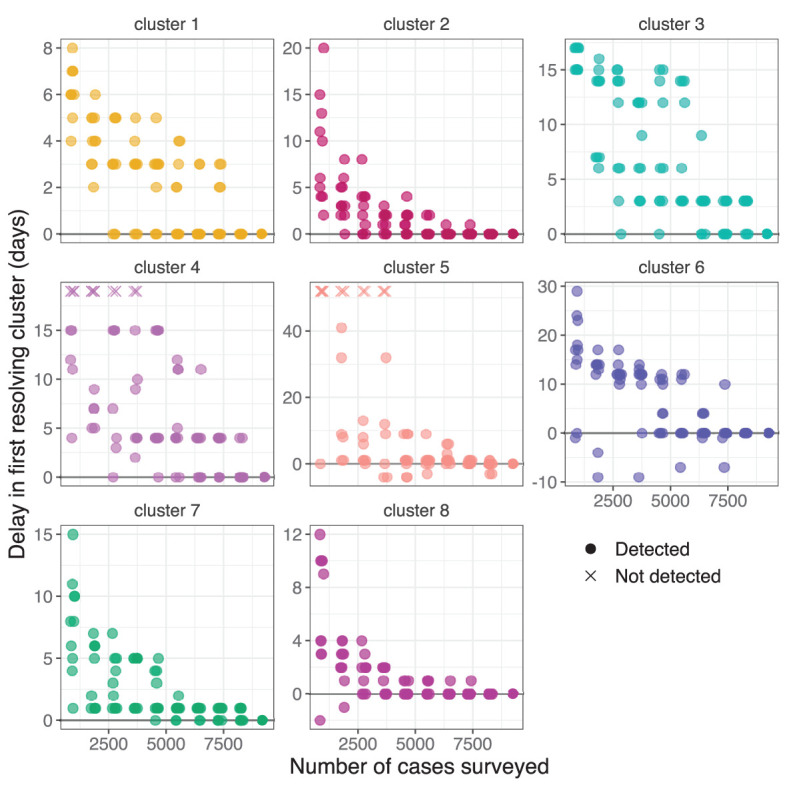
Delay in first resolving each cluster from the NSW Delta variant outbreak when selecting different random subsets. The vertical axis show the delay in first resolving each cluster compared to the full dataset. Subsets where a cluster was not detected are indicated with a cross at an arbitrary vertical position. Transparency and a small random horizontal spread are added for visibility of duplicate points.

Samples of different sizes were drawn from the full set of cases without replacement. For each sample, we performed clustering for each day using the nearest neighbor method with *k* = 4 and the threshold method with thresholds 1 SNP and 7 days. We recorded the first day at which each genomic cluster was identified by at least one informative cluster, and subtracted the corresponding value from the full dataset to measure the relative delay in first resolving the cluster (Figure 7). Identification of the largest cluster, cluster 1, was delayed by up to a week at very low surveillance depths, and other clusters were delayed by 1–3 weeks. The delay tended to increase as the proportion of cases sampled decreases, although there was a large spread in the measured delays particularly in smaller clusters such as clusters 2 and 7. In some samples the delay was negative, meaning that the cluster was identified at an earlier date than was possible with our method when using the full dataset.

## 4. Discussion

The COVID-19 pandemic has highlighted the role of genomic surveillance in public health responses but also the challenges of surveillance related to sampling biases and sustained implementation. Genomic surveillance has been instrumental to enable understanding of the continuous genetic evolution and spread of SARS-CoV-2 variants but, at the same time, has suffered from a lack of systematic methodological approaches for optimisation in the context of rapidly changing public health needs. Our findings illustrate an approach to estimate the performance of SARS-CoV-2 genomic surveillance by quantifying the expected loss of clustering resolution in an outbreak setting as the depth of surveillance is reduced. This can be used to inform the design of genomic surveillance programs for different pathogens by adapting the simulation parameters and scenarios. Our findings extend existing literature on estimating the rate of under-reporting during outbreaks and the effect on estimators of outbreak parameters (11, 33, 34), and guidelines for the design of genomic surveillance programs (1, 5, 16).

An important strength of our method is that both clustering methods are based on arbitrary dissimilarity measures and as such can be extended to include other data types. For example, if spatial information about cases is available then it can be incorporated as an additional threshold into the threshold method, and the spatial neighborhood could be included in the *k*-neighborhood for the nearest neighbor method. Similarly, genetic dissimilarity could be measured using metrics based on phylogeny or a pathogen-specific model of mutation instead of the SNP distance in order to better reflect the methods used for outbreak investigation.

### 4.1. Clustering algorithms

In our method, the determination of whether an outbreak would be successfully detected is automated by means of two clustering algorithms. The threshold method has previously been proposed (23). The nearest neighbor method draws from the principles of an established test for interaction between distances in different data types (24) and the proposed use of the implied links between cases for visual diagnostics of the test (35). To our knowledge, its adaptation as the basis of a clustering algorithm is novel. The combination of the two clustering methods is designed to simulate the likelihood of success of a conventional analysis rather than to exhaustively cluster cases.

The clustering algorithms used in this study have parameters which must be specified and which can potentially have a large impact on their performance. For the threshold method, the choice of thresholds can be made based on assumed distributions of outbreak parameters such as serial intervals and mutation rates, or chosen to maximize the number of informative clusters. The nearest neighbor method requires a choice of *k* to be specified. Small values of *k* will tend to produce small clusters which may increase the chance of providing informative clusters at the expense of splitting outbreaks, while large values are more likely to merge outbreaks. In practice, the choice of *k* = 4 often produces reasonable clusters as seen in Figure 2. As the nearest neighbor method can be interpreted as a hierarchical clustering algorithm, there is a possibility to extend the method to dynamically select clusters of different *k* values as is done in other hierarchical algorithms such as HDBSCAN (36). Other algorithms for outbreak detection that combine multiple data types have previously been published (37).

### 4.2. Performance under reduced depth of surveillance

When the proportion of cases undergoing WGS decreases, the ability to resolve clusters is impacted in several ways. There is a chance that a given subset of cases will include few or no cases from the specific outbreak. This effect is modeled by the ECDC in its guidance for representative sampling for genomic surveillance (5) and is the main reason for the difficulty in identifying small outbreaks. When surveillance is reduced, intermediate cases in transmission chains are missed. If the missed links are randomly distributed, this results in a greater average distance between cases, however if several consecutive cases along a transmission chain are missed, the outbreak can be split into multiple clusters. If these split clusters are too small or are clustered with unrelated cases, then this will result in uninformative clusters.

The threshold method is susceptible to cases bridging multiple outbreaks. This can be seen in the largest graph component in Figure 2C which has clear substructure with a single case from one outbreak bridging cases from three other outbreaks. The same phenomenon is responsible for the fact that the algorithm's performance can counter-intuitively degrade with better surveillance coverage as seen in the upper-right-most panel of Figure 5. With all cases included, some large outbreaks are bridged and therefore not resolved, whereas when the bridging cases are missed, the outbreaks are clustered separately and therefore resolved. An extension using graph structure other than connected components to define the clusters could improve the algorithm's robustness.

The nearest neighbor method is less likely to cluster multiple outbreaks together, particularly at smaller values of *k*. In Figure 2D, the method provided several smaller informative clusters for cases from outbreak 9 which were instead merged by the threshold method, and the largest uninformative cluster contains cases from only three outbreaks compared to four in the threshold method. This effect is most important when the genomic similarity between outbreak sequences is high. In Figure 5, scenarios with lower values for both the ancestral divergence time and index case window (corresponding to more similar genome sequences) had a larger proportion of simulations where only the nearest neighbor method detected the outbreak.

### 4.3. NSW SARS-CoV-2 outbreaks in 2020–2021

In NSW the SARS-CoV-2 pandemic arrived in three small waves in 2020–2021 followed by the significantly larger Delta variant wave beginning in mid 2021. Waves 2 and 3 of the pandemic in 2020 were characterized by small local clusters of transmission (38, 39). Almost all cases in both waves were epidemiologically linked, reflective of limited community transmission, little of which went undetected. In these early waves, the low genetic diversity of locally-transmitted cases implies that essentially any level of genomic surveillance would have succeeded in identifying a representative sample of the diversity of the outbreak. Other than for monitoring diversity, WGS had important roles in establishing that the outbreak strain was a recent importation as opposed to one that had been circulating undetected, and in supporting epidemiological investigation.

The subsequent Delta variant outbreak, by contrast, led to sustained community transmission and distinct genomic sub-clusters emerging after several weeks of circulation. The detection of these sub-clusters improved the perceived utility of WGS for resolving contentious epidemiological links, and allowed for phylogeographic analysis to support public health decision making. We found that under reduced depth of surveillance the identification of these sub-clusters would have been delayed by several days (Figure 7).

### 4.4. Limitations

Several limitations of this study have to be acknowledged. Firstly, we assess outbreak detection using our definition of informative genomic clusters, which differs from typical approaches to detecting outbreaks through epidemiological investigation. This is necessary because our approach requires efficient and automatic assessment of many scenarios and so does not incorporate the broader, context-specific range of evidence used by public health professionals.

Secondly, the outbreak simulation used in our comparisons of scenarios are based on a simple branching process and genome model that do not capture some potentially significant contributors to disease incidence such as host population dynamics and molecular evolution. While the scenarios we present here involve relatively short time scales, more sophisticated modeling would be necessary to generate realistic SNP distance profiles over longer periods. We chose to select outbreaks that became extinct within the simulation window, which may not be representative of the transmission dynamics of some pathogens and constrains the reproduction number distribution. Our method accepts pairwise distance data and can therefore be applied to data generated using alternative simulations.

Thirdly, our findings use a model of genomic surveillance where cases are expected to be sampled uniformly at random, however this is rarely possible in practice as previously noted. This is not a fundamental limitation of our method, as arbitrary selection strategies can be accommodated straightforwardly.

Lastly, part of our findings are based on local data from NSW and simulation settings compatible with the Delta lineage, and may not generalize to other contexts. We intend the clustering configuration, simulation details, and sampling strategies to be adjusted and validated for the local context in order to provide relevant guidance for genomic surveillance.

### 4.5. Conclusions

Previous recommendations for SARS-CoV-2 genomic surveillance have focused on the minimum sample size required to ensure that an emerging variant is detected with good confidence given a rate of reported cases in the community and the expected prevalence of the new variant. Our method is instead based on the identification of emerging clusters using automated clustering techniques to emulate outbreak investigation. We therefore see our approach as providing complementary evidence to inform the choice of sampling strategy where additional resolution is a key objective of the surveillance system. With the increasing importance of genomic epidemiology in public health responses, we anticipate a growing need to accommodate more sophisticated applications of genomic data. The design of genomic surveillance programs will subsequently require more nuanced evidence to ensure adequate resources are allocated to achieve the public health outcomes of the programs.

## Data availability statement

The code necessary to reproduce the outbreak simulation data and quantitative results from this study is available at https://doi.org/10.5281/zenodo.6860216. A list of the SARS-CoV-2 genome sequences and pairwise distances used in the analysis of the NSW outbreak is available at https://doi.org/10.5281/zenodo.6860154. Genome sequences are available from the GISAID repository via https://gisaid.org/EPI_SET_220919ef.

## Ethics statement

The studies involving human participants were reviewed and approved by Western Sydney Local Health District Human Research Ethics Committee (2020/ETH02426). Written informed consent from the participants' legal guardian/next of kin was not required to participate in this study in accordance with the national legislation and the institutional requirements.

## Author contributions

Study concept and design by CS, AA, and VS. Data generation, collection, and analysis by AA, GB, MG, JD, EM, AD, and RR. CS developed the methodology, curated data for analysis, and wrote analysis code. Study coordination by SC, JK, DD, and VS. CS wrote the first manuscript draft, with editing from AA and VS. All authors read and approved the final manuscript.

## Funding

This study was supported by the Prevention Research Support Programme funded by the New South Wales Ministry of Health. This research was partially supported by the Australian Government through the Australian Research Council's Discovery Projects funding scheme (project DP220101688).

## Conflict of interest

The authors declare that the research was conducted in the absence of any commercial or financial relationships that could be construed as a potential conflict of interest.

## Publisher's note

All claims expressed in this article are solely those of the authors and do not necessarily represent those of their affiliated organizations, or those of the publisher, the editors and the reviewers. Any product that may be evaluated in this article, or claim that may be made by its manufacturer, is not guaranteed or endorsed by the publisher.
