# Supplementary material for: Guiding the design of SARS-CoV-2 genomic surveillance by estimating the resolution of outbreak detection
Source: Front Public Health. 2022 Oct 5;10:1004201. doi: 10.3389/fpubh.2022.1004201 (PMC9581317; doi:10.3389/fpubh.2022.1004201)
Supplement: Supplementary file 1 [file Data_Sheet_1.PDF]

Supplementary Material

Guiding the design of SARS-CoV-2 genomic surveillance by estimating the resolution of outbreak detection

Carl J.E. Suster, Alicia Arnott, Grace Blackwell, Mailie Gall, Jenny Draper, Elena Martinez, Alexander P. Drew, Rebecca J. Rockett, Sharon C.-A. Chen, Jen Kok, Dominic E. Dwyer, and Vitali Sintchenko

1 SIMULATION DETAILS

1.1 Branching process

A branching process is used to generate cases. The incubation time for each case is drawn from a Poisson distribution with rate $\lambda = 2$. A case's infectiousness is the product of (1) the relative infectiousness which is 1 on the day that the incubation period ends and decreases to almost zero by day 10 and (2) an individual reproduction number drawn from a gamma distribution with shape $k = 2.5$ and scale $\theta = 0.3$. The relative infectiousness t time steps after infection is $2 \times p(10, t + 10)$ where $p(\lambda, k) = \lambda^k e^{-\lambda}/k!$, an approximate fit to reported infectiousness curves. The distribution for the individual reproduction number affects both the total number of cases generated and the shape of the outbreak. The mean value of the chosen distribution is $k\theta = 0.75$, resulting in contained outbreaks consistent with strong public health measures.

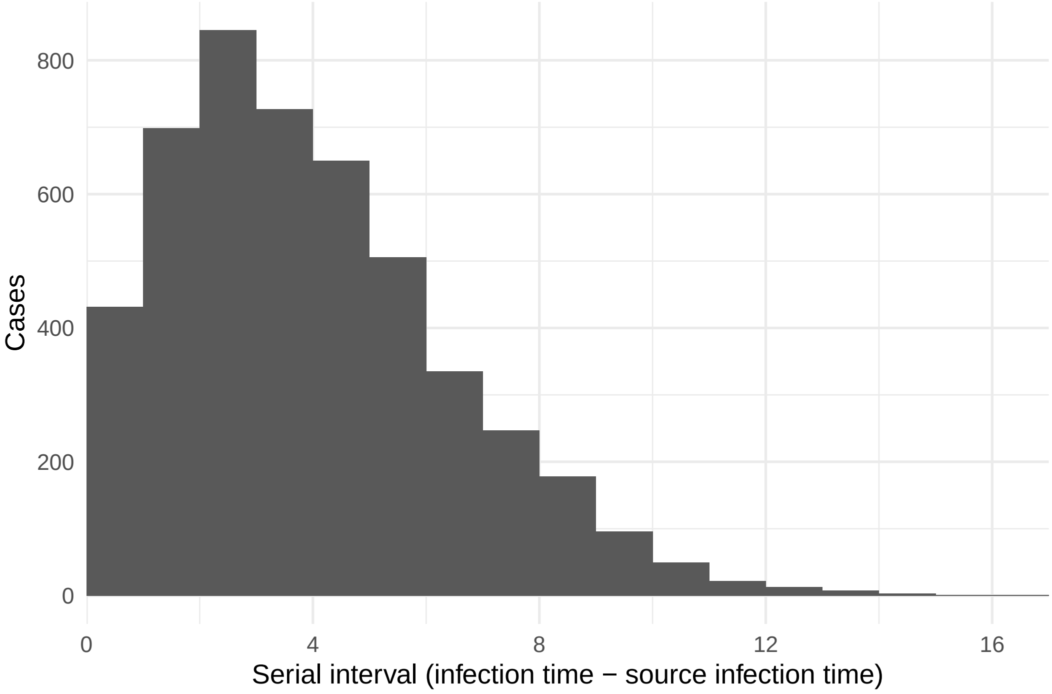

Figure S1. Histogram of the serial interval for the baseline simulation configuration. The distribution is broadly compatible with reported values, noting that the simulation is simple and does not attempt to model detailed features driving transmission dynamics.

At each time step of the process, new cases are generated according to a Poisson distribution with the rate set to the sum of the infectiousness of all existing cases. New cases are assigned a source from the existing cases weighted by their infectiousness. The process is terminated when the total infectiousness becomes negligible, or if the cumulative number of cases generated at a time step exceeds the configured maximum (in which case the result is discarded). The serial interval is shown in Figure S1. The reporting date for each case is set as the day that the incubation period ends with a delay added according to a Poisson distribution with rate $\lambda = 1$. The pairwise temporal distance is taken as the number of days between the reporting dates.

1.2 Genome model

Genomes are represented by a list of 64 positions with two states, stored compactly within 8 bytes. The genome associated with a case is generated from its source case's genome by flipping at random a number of positions (bits) determined by a Poisson distribution with rate proportional to the expected mutation rate and the serial interval. The total number of mutations per day is approximated as $(1.1\times10^{-3}$ nt^{-1} year$^{-1})$ $\div$ (365 day $\cdot$ year^{-1}) $\times$ (30000 nt). The genome size is accounted for in the expected mutation rate such that the finite width of the genome's representation does not affect the simulated rate of mutations, only the likelihood of mutations occurring at sites that have previously acquired a mutation. The pairwise genetic distance is the number of positions that differ in the genome representation.

2 SIMULATION PARAMETER VARIATIONS

Results for several variations of the simulation parameters (Table S1) are summarised in Figure S2. Scenario A is the configuration presented in the main text for reference. Scenarios B and C model a situation where the goal is to distinguish medium sizes clusters from a background of unclustered cases. For scenario C, the average genetic distance amongst the unclustered cases and between the unclustered cases and the index cases of the outbreaks are all decreased, and consequently a lower proportion of outbreaks are identified compared to scenario B. In both scenarios B and C the nearest neighbour method fails to identify the outbreaks. Scenario D halves the mutation rate compared to scenario A resulting in increased genetic similarity between cases and consequently a lower proportion of outbreaks identified. Scenarios E and F show variations of the clustering algorithm parameters. In scenario E, a smaller value of k has little effect on the performance of the nearest neighbour method compared to scenario A. Note that larger values of k tend to result in poorer performance as outbreaks are merged into larger clusters. In scenario F the genetic and temporal distance thresholds are increased resulting in poorer performance by the threshold method.

Table S1. Parameter definitions for simulation scenarios in Figure S2.

	Outbreak size			Singletons	Mutation rate	Ancestral divergence	k	Thresholds	
	small	medium	large		(nt^{-1} year^{-1})	time (days)		SNPs	days
A	4	3	2	20	1.1×10^{-3}	7	4	1	7
B	0	4	0	1000	1.1×10^{-3}	7	4	1	7
C	0	4	0	1000	1.1×10^{-3}	0	4	1	7
D	4	3	2	20	5.5×10^{-4}	7	4	1	7
E	4	3	2	20	1.1×10^{-3}	7	3	1	7
F	4	3	2	20	1.1×10^{-3}	7	4	2	14

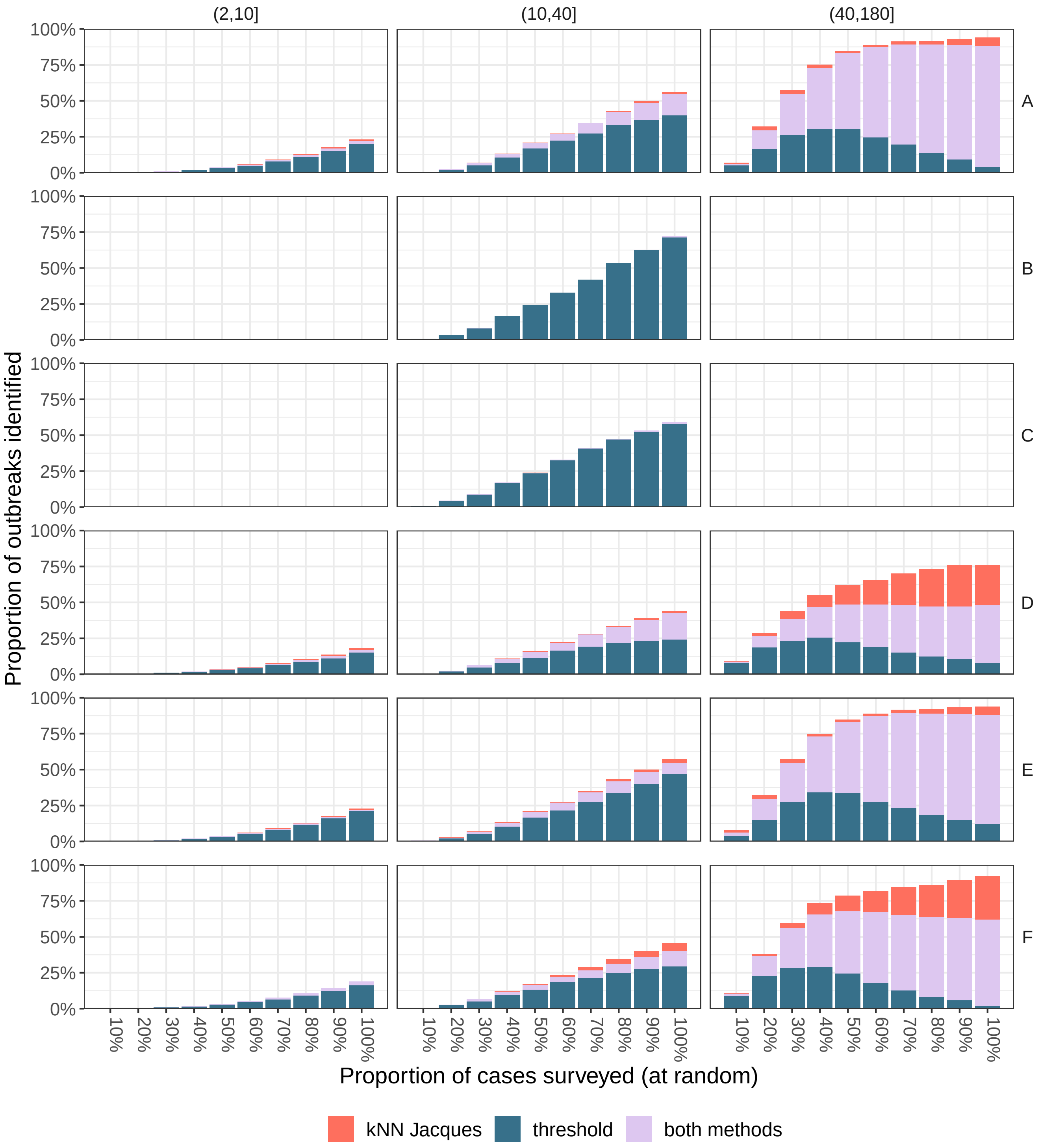

Figure S2. Resolution of outbreaks at $t_{25\%}$ for several variations of modelling parameters as outlined in Table S1. The 3 columns correspond to the size of the outbreaks measured by the total number of simulated cases. The colour of the bars indicates whether one or both clustering methods resolved the outbreaks. The proportion of outbreaks identified is averaged over 25 different simulations and 50 different subsets for each proportion of cases surveyed.

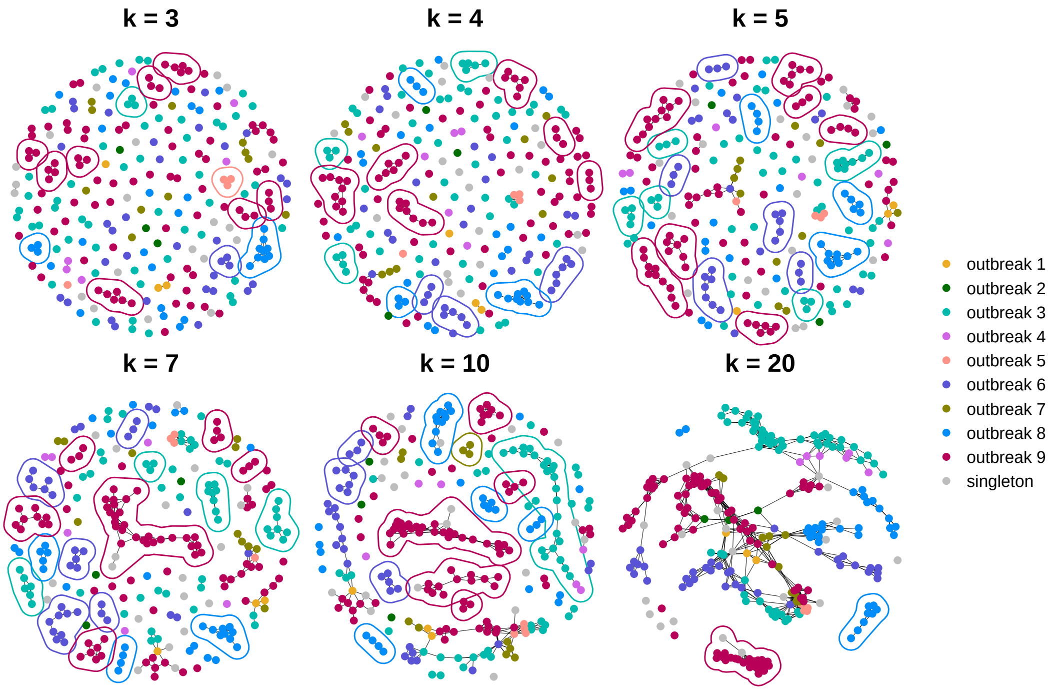

Figure S3. Clusters generated by the nearest neighbour method with different choices of k from the same simulation shown in Figure 2.

3 CHOICE OF k IN NEAREST NEIGHBOUR METHOD

The nearest neighbour clustering method requires a choice of k. The effect of increasing k is illustrated in Figure S3: at smaller values clusters tend to be small, and many points are not clustered, whereas at larger values of k, large clusters are formed containing several outbreaks. The large connected components in the graphs for $k = 10$ and $k = 20$ have clear substructure corresponding to outbreak membership, with more edges between cases in the same outbreak than inter-outbreak edges.

Note that the method creates edges between a pair of nodes when one is in the *intersection* of the k nearest neighbour sets of the other corresponding to each distance measure. Therefore if more than two distance measures are used, it is likely that fewer edges will be created and therefore that the clusters will be smaller for the same choice of k.

As we use the nearest neighbour method concurrently with the threshold method, we prefer a value of k that results in good detection of outbreaks in scenarios where the threshold method performs poorly. These scenarios correspond to the panels of Figures 4 and 5 with the largest orange segments, i.e. where the index cases are most similar or the proportion surveyed is lowest.

For the scenarios in Figure 4, the effect of changing k is summarised in Figure S4. As k was increased, the nearest neighbour method detected fewer outbreaks overall, and by $k = 10$ it was detecting almost no outbreaks that were not already detected by the threshold method. There was minimal difference in performance between $k = 3$ and $k = 5$.

For the scenarios in Figure 5, the effect of changing k is summarised in Figure S5. As above, the nearest neighbour algorithm performed poorly at high values of k, and there was minimal difference in performance between $k = 3$ and $k = 5$.

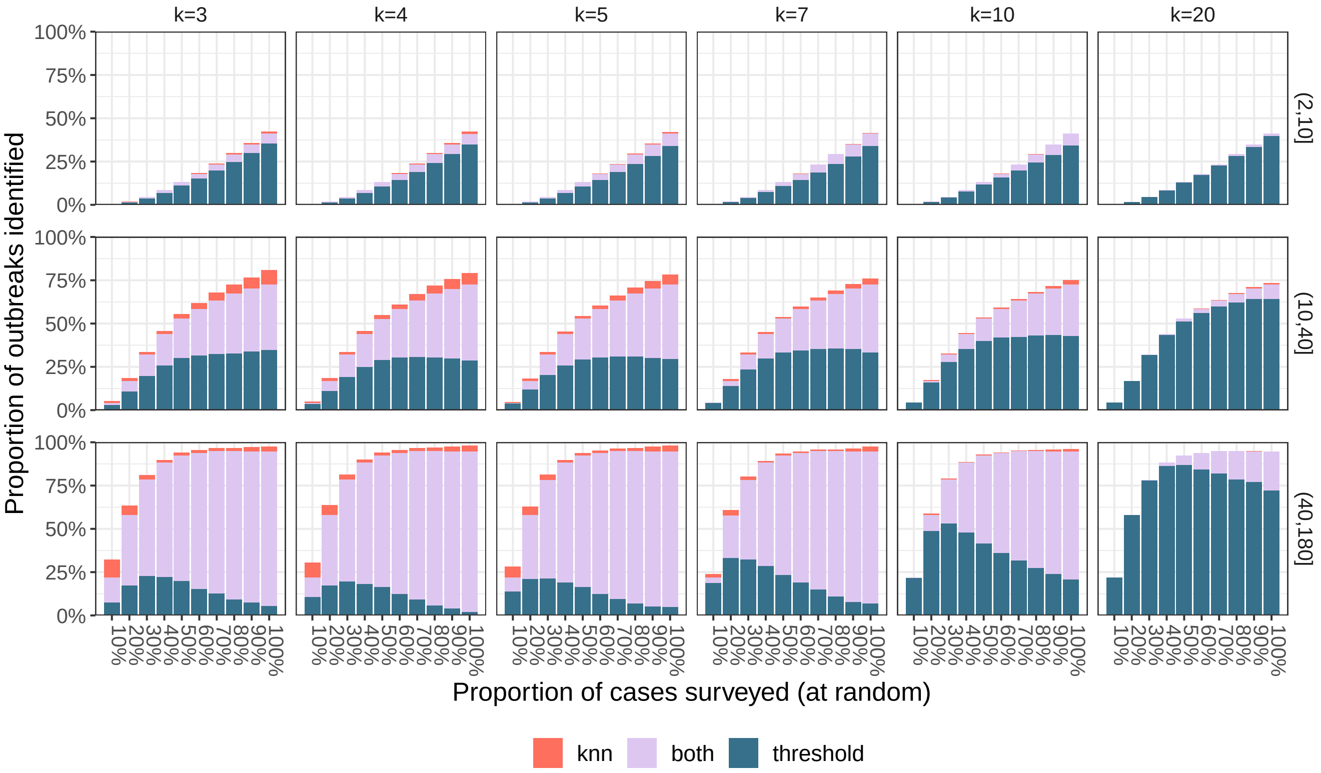

Figure S4. Resolution of outbreaks at $t_{25\%}$ for different choices of k. The column where $k = 4$ corresponds to the first column of Figure 4.

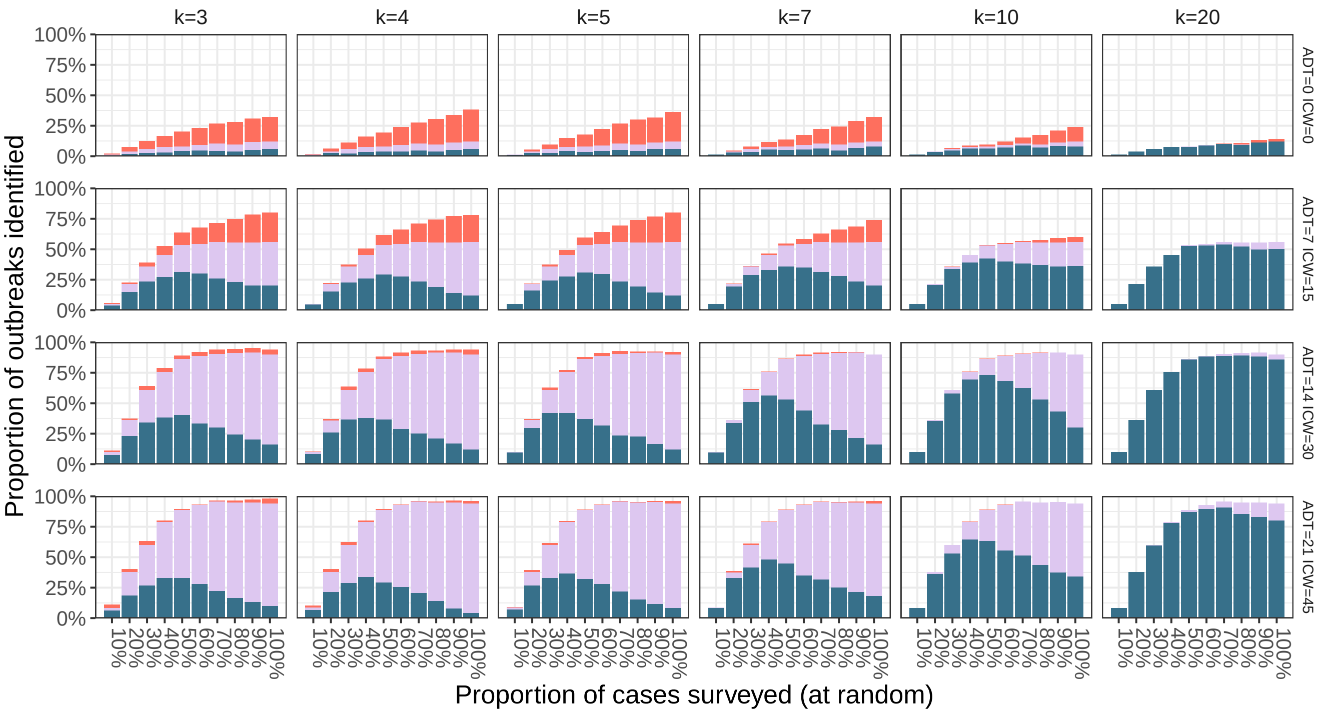

Figure S5. Resolution of large outbreaks at $t_{25\%}$ for different choices of k. The column where $k = 4$ corresponds to the diagonal panels of Figure 5.

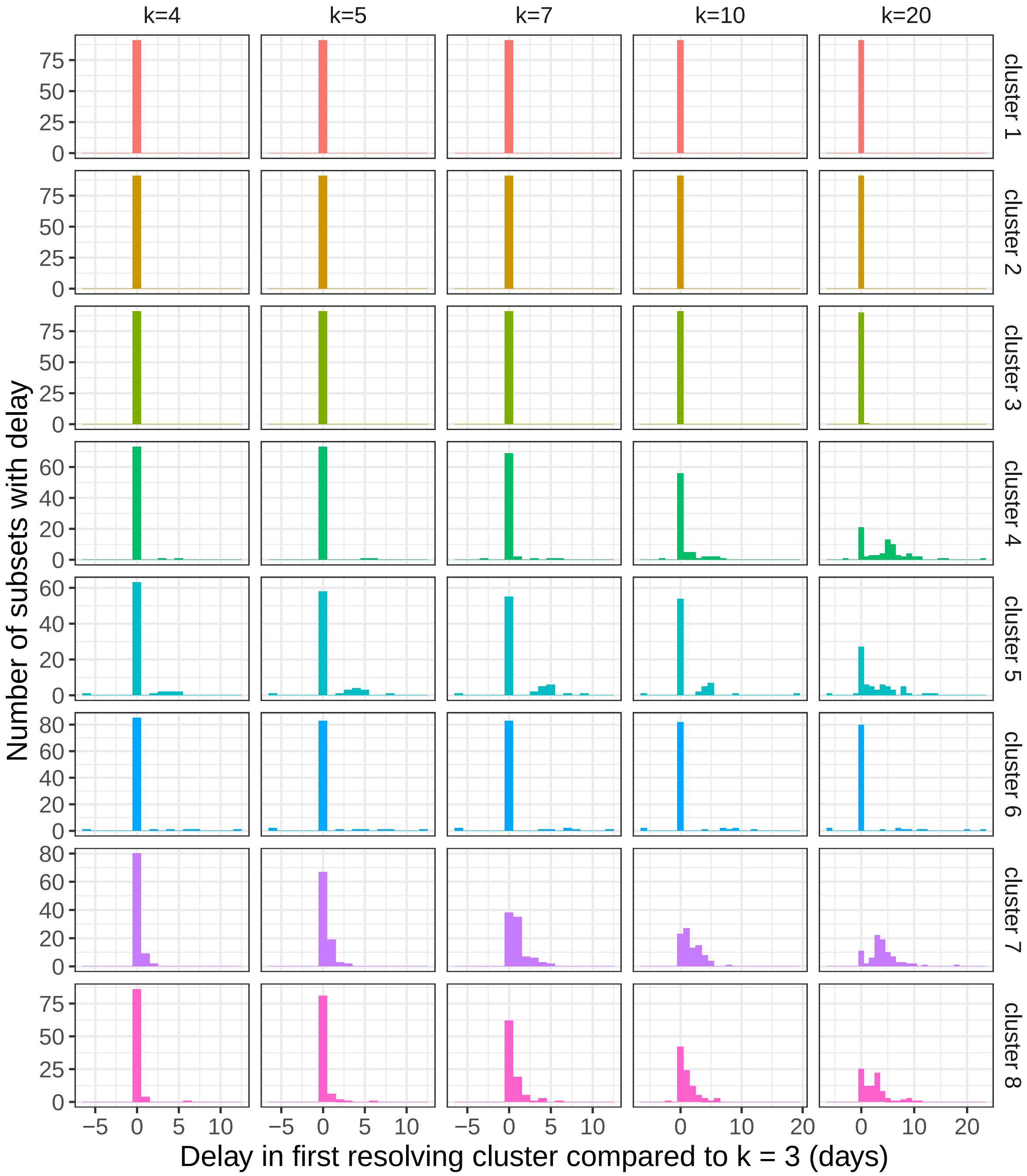

Figure S6. Delays in detecting clusters in the NSW Delta variant outbreak compared to $k = 3$.

For the NSW Delta variant outbreak, increasing k leads to a greater chance of each cluster being detected later. Figure S6 shows the detection delays compared to $k = 3$ for the same subsets (of different sizes) of the data used in Figure 7. For $k > 5$, the detection of clusters 7 and 8 was delayed in most subsets. For $k = 4$ and $k = 5$, the first detection of all clusters occurred on the same day as for $k = 3$ in almost all subsets.

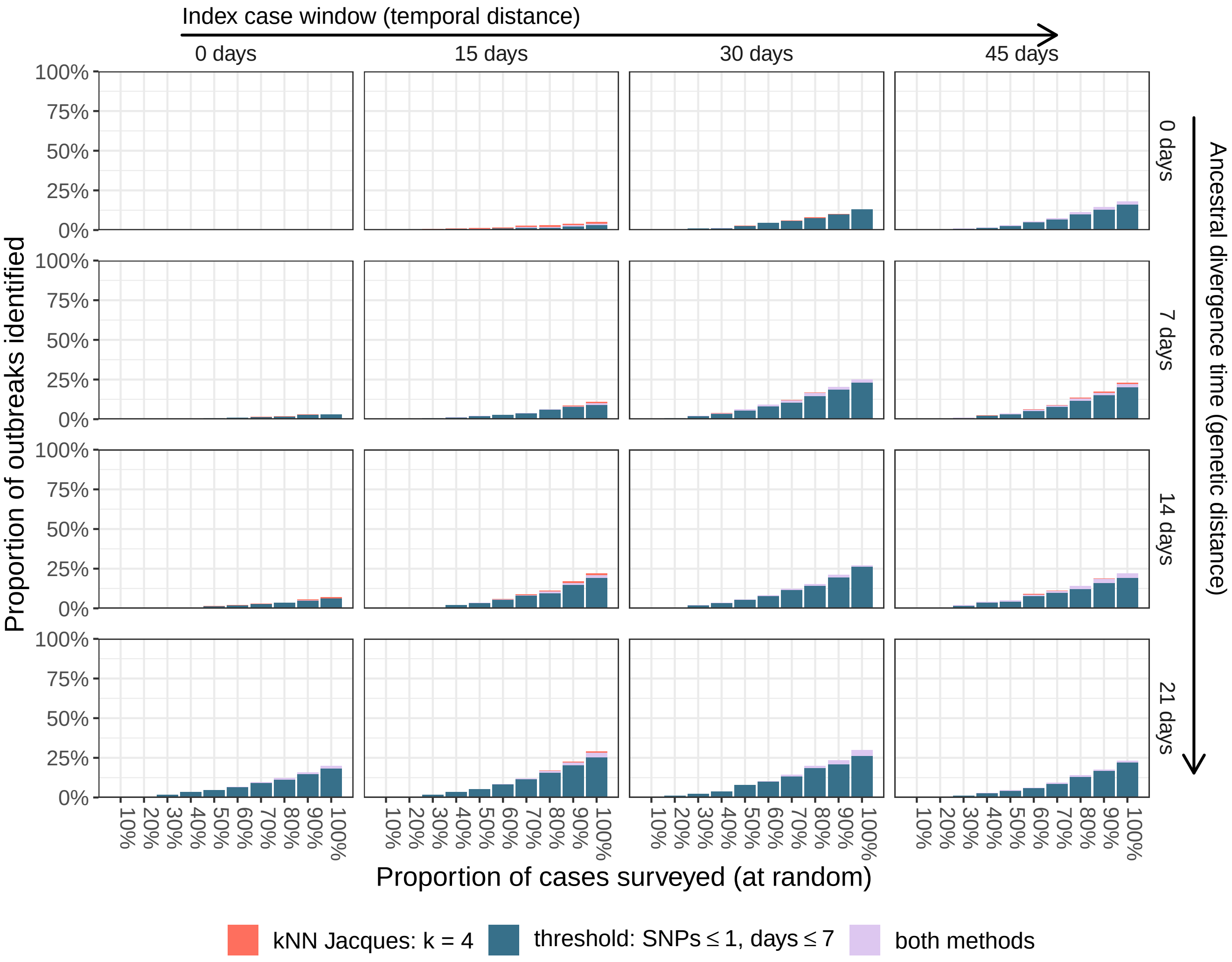

Figure S7. Resolution of small outbreaks at $t_{25\%}$ as the index case window and the ancestral divergence time are varied. The simulations are the same as in the equivalent figure for large outbreaks in the main text. The colour of the bars indicates whether one or both clustering methods resolved the outbreaks. The proportion of outbreaks identified is averaged over 25 different simulations and 20 different subsets for each proportion of cases surveyed.

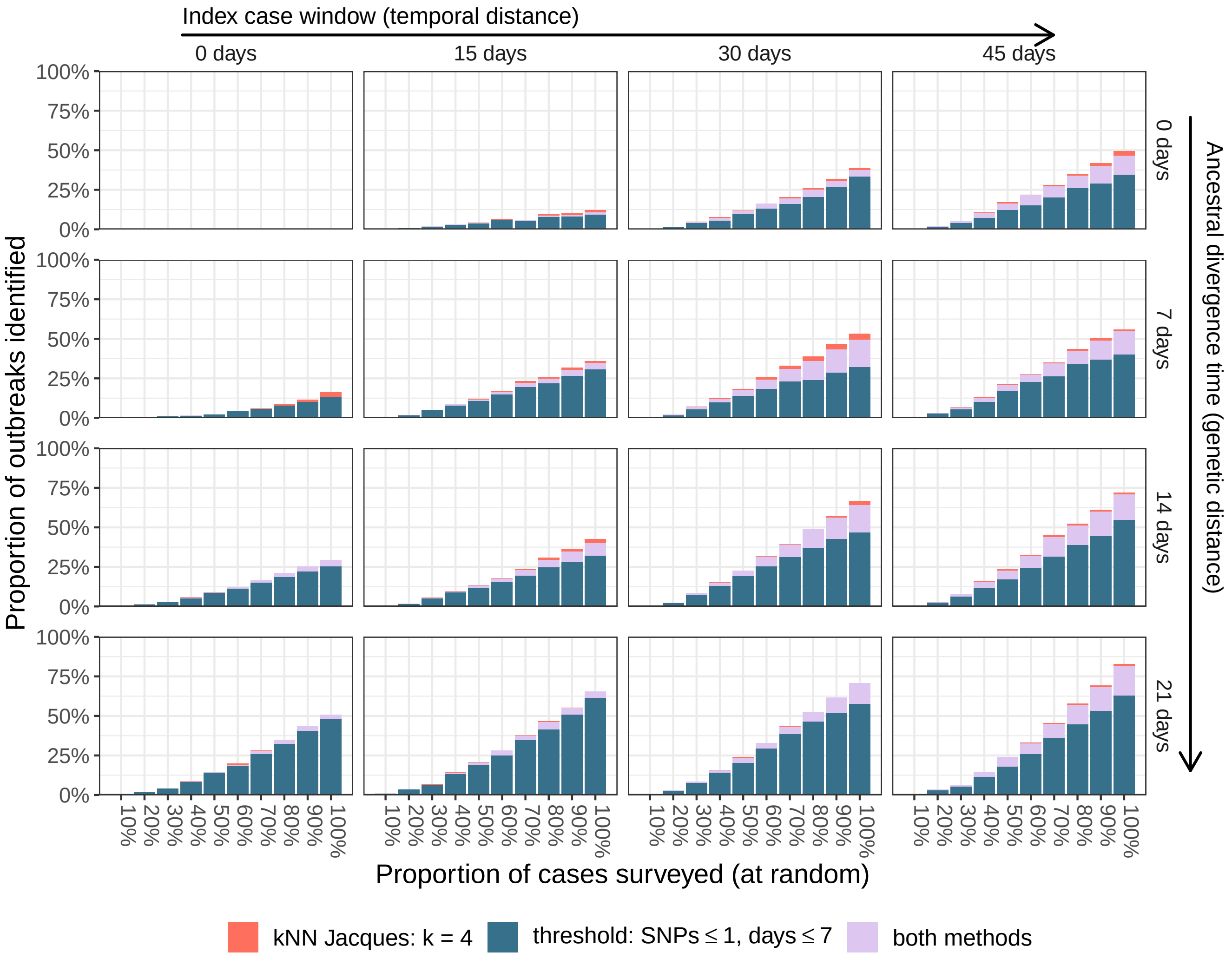

Figure S8. Resolution of medium outbreaks at $t_{25\%}$ as the index case window and the ancestral divergence time are varied. The simulations are the same as in the equivalent figure for large outbreaks in the main text. The colour of the bars indicates whether one or both clustering methods resolved the outbreaks. The proportion of outbreaks identified is averaged over 25 different simulations and 20 different subsets for each proportion of cases surveyed.

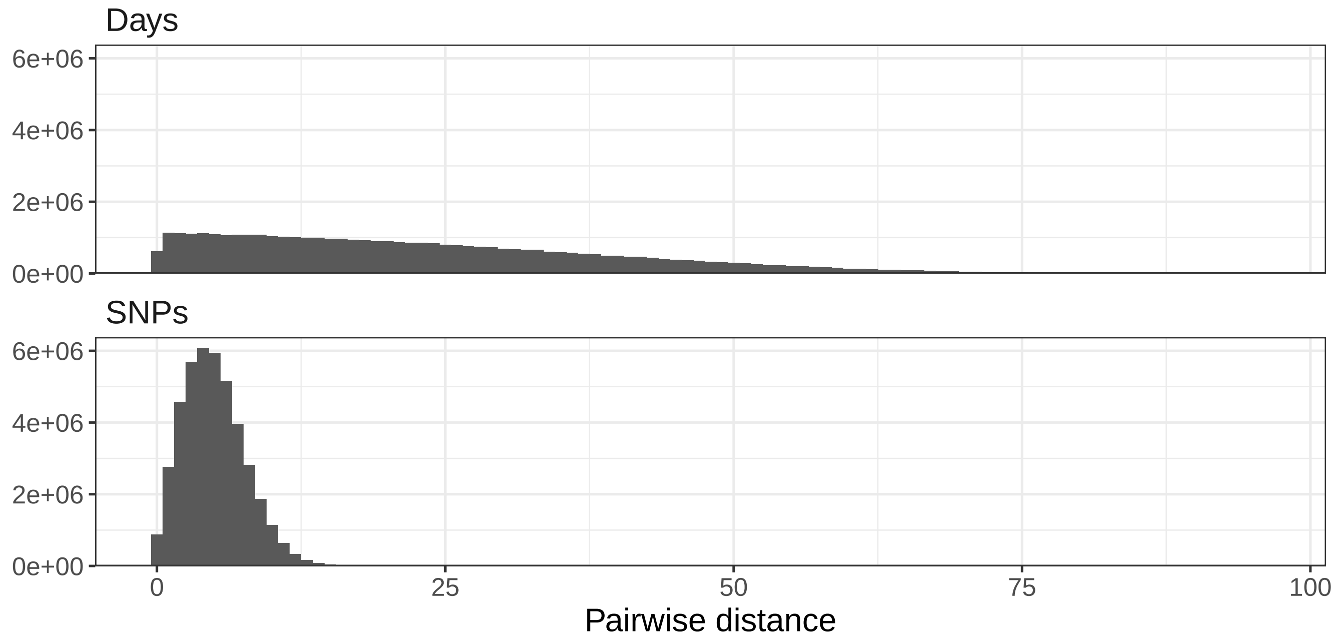

Figure S9. Histograms of the pairwise distances between cases in the NSW Delta outbreak by the date of collection (top) and SNP distance (bottom).
